# Case report of *Ureaplasma urealyticum* meningitis in a patient with thymoma and hypogammaglobulinaemia

**DOI:** 10.1186/s12879-021-06831-z

**Published:** 2021-11-08

**Authors:** Ting Zhang, Haiyan Li, Shuping Hou, Huanxin Yu, Wei Yue

**Affiliations:** 1 Clinical College of Neurology, Neurosurgery and Neurorehabilitation, Tianjin Medical University, Tianjin, China; 2 Present Address: Department of Neurology, Tianjin Key Laboratory of Cerebrovascular and Neurodegenerative Diseases, Tianjin Huanhu Hospital, Tianjin, China; 3 Department of Otolaryngology, Tianjin Key Laboratory of Cerebrovascular and Neurodegenerative Diseases, Tianjin Huanhu Hospital, Tianjin, China; 4 Department of Dermatology, Tianjin Medical University General Hospital, Tianjin, China

**Keywords:** *Ureaplasma urealyticum*, Meningitis, Thymoma, Hypogammaglobulinaemia

## Abstract

**Background:**

*Ureaplasma urealyticum* (UU) is found among the normal vaginal flora in a considerable proportion of asymptomatic women; however, adult central nervous system (CNS) infection of UU is extremely rare. Good's syndrome (GS) is an adult-onset immunodeficiency characterized by thymoma, hypogammaglobulinaemia, low or absent B‑cells, and an inverted CD4+/CD8+ T‑cell ratio. Patients with GS usually have severe or recurrent infections.

**Case presentation:**

We describe the case report of a 49-year-old woman who developed UU meningitis. Initial routine anti-viral and anti-bacterial therapy showed no improvement in the patient's condition. Next-generation sequencing (NGS) of cerebrospinal fluid (CSF) identified the UU DNA sequence. Accordingly, a diagnosis of UU meningitis was made, and minocycline therapy was initiated. The patient responded favourably, with no signs of disease at subsequent follow-up. According to the severity and rarity of the case, secondary immunodeficiency was suspected. Flow cytometry found hypogammaglobulinaemia. Combined with the previous history of thymoma, the patient was diagnosed with immune deficiency disease of GS.

**Conclusions:**

This case may be the first adult case report in the literature describing UU meningitis in a patient with GS. The diagnosis of GS should be considered in patients presenting with unexplained antibody deficiency and thymoma.

## Background

Meningitis caused by *Ureaplasma urealyticum* (UU) is a rare disease. UU, a member of the Mycoplasma family, is the smallest prokaryotic cell type microorganism that can pass through a filter and grow in an inanimate medium. The microorganism has no cell walls and is very vulnerable to drying and other environmental exposures. UU can attach to different types of epithelial cells and grow mainly on the mucosal surface in the genitourinary tract of adults or in the respiratory tract of infants. The available data show that the host's innate immune response plays a decisive role in the manifestation of UU infection [1].

In 1954, Robert Good, who played a crucial role in modern immunology, first reported thymoma with hypogammaglobulinaemia (Good's syndrome, GS) and found that approximately 10% of patients with hypogammaglobulinaemia were often diagnosed with thymoma. GS patients have a kind of T cell that inhibits gamma globulin synthesis. However, the number of T cells in circulating blood in most of these patients is still within the normal range [2]. It is essential to point out that GS is a combined immunodeficiency (humoral and cellular) that is now classified as an entity separate from common variable immunodeficiency disease (CVID). Unlike patients with CVID, GS patients usually present with opportunistic infections [3].

In the present study, we report a case of UU meningitis in a patient with thymoma and hypogammaglobulinaemia. Adult central nervous system (CNS) infection of UU is sporadic. To our knowledge, although UU meningitis has been reported in patients with hypogammaglobulinaemia, it has not been reported in a patient with GS.

## Case presentation

On June 30, 2018, a 49-year-old woman was admitted to our hospital. Two weeks prior, she underwent functional endoscopic sinus surgery at an ear, nose, and throat (ENT) clinic due to sinusitis. The day after the surgery, she developed a headache, which continued to worsen and was accompanied by nausea and vomiting. She was then admitted to the hospital through the Neurology Emergency Department. Her headache was located frontally, parietally, and occipitally. The visual analogue scale (VAS) score was eight on admission. Neurological examination showed no focal deficits except neck stiffness. Body temperature was 37.4 °C. Ten years earlier, the patient had undergone resection for a pathologically confirmed type B1 thymoma that was accidentally found by chest computed tomography (CT) examination in the outpatient clinic due to cough symptoms.

**Fig. 1:**
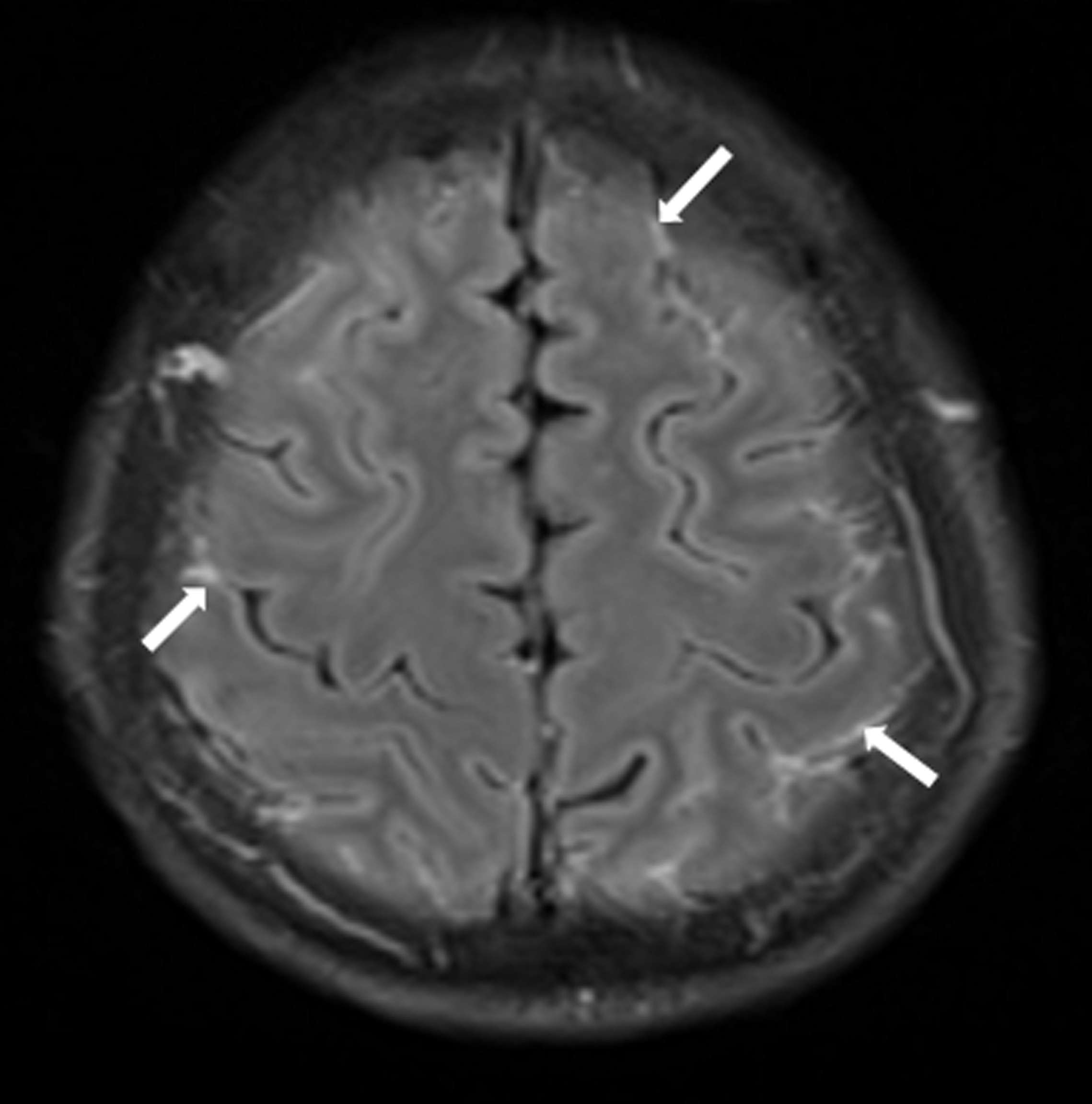
Magnetic resonance image of the brain. Abnormal hyperintensity in the cerebral sulci of the bilateral frontal-parietal lobes can be observed on fluid-attenuated inversion recovery sequences with gadolinium enhancement

Head CT showed no abnormality, but inflammation of the paranasal sinuses was observed in the whole bilateral group. Magnetic resonance imaging (MRI) demonstrated abnormal hyperintensity in the cerebral sulci of the bilateral frontal-parietal lobes on a fluid-attenuated inversion recovery sequence with gadolinium enhancement (Fig. 1).

The first lumbar puncture was completed on the day of admission, which revealed 24 mmHg opening pressure. Cerebrospinal fluid (CSF) examination revealed the following: reduced glucose (0.60 mmol/L) and chlorine (116 mmol/L), increased protein (1.01 g/L), and leukocytosis (740 × 10^6^/L; multinucleate cells, 82%), which suggested an infectious aetiology. Routine CSF microbiological examinations (Gram stains, acid-fast stains, ink stains) were negative, and cultures for bacteria (i.e., aerobic bacteria: *Escherichia coli*, *streptococci*, *staphylococci*; anaerobes: *Bacillus perfringens*, *Bacteroides fragilis*) and fungi (i.e., *Cryptococcus neoformans*) were sterile. Leukopenia (white blood count, 1.93*10^9^/L) and neutropenia (neutrophil count, 0.81*10^9^/L) were noted. Serological testing for human immunodeficiency virus (HIV) was negative; anti-neutrophil antibodies (ANCAs) were undetectable. Tests for HIV and ANCA are administered routinely to patients who present with unexplained infection. Intravenous ganciclovir (300 mg i.v. twice a day [q12 h.]), fluconazole (200 mg i.v. once a day [q.d.]), levofloxacin (500 mg i.v. once a day [q.d.]), ceftriaxone (2000 mg i.v. once a day [q.d.]), and vancomycin (1000 mg i.v. twice a day [q12 h.]) were administered empirically but without improvement.

The second lumbar puncture was performed on July 8 and revealed a 23 mmHg opening pressure. CSF examination showed reduced glucose (0.91 mmol/L), increased protein (1.35 g/L), and leukocytosis (300 × 10^6^/L; multinucleate cells, 74%). CSF was sent for next-generation sequencing (NGS). Five days later, UU DNA sequences were identified by NGS analysis; the number of identified sequence reads was 38,289. We added minocycline (100 mg p.o. twice a day [b.i.d.]) to the patient’s therapeutic regimen immediately, as minocycline is particularly efficacious against UU and accumulates at a high concentration in CSF. We retained levofloxacin (500 mg i.v. once a day [q.d.]) and stopped all the other anti-infective treatments (ganciclovir, fluconazole, ceftriaxone, and vancomycin). On July 14, to locate the source of infection, we took a small amount of paranasal sinus mucosa through nasal endoscopy for NGS, and UU was also found (reads were 98). Polymerase chain reaction (PCR) and Sanger sequencing confirmed the UU gene in both CSF and paranasal sinus mucosa samples. The patient's headache improved markedly. On July 19, a repeat lumbar puncture revealed decreased opening pressure (5 mmHg). On CSF examination, cell counts and protein levels were reduced to 46 × 10^6^/L and 0.61 g/L, respectively, whereas glucose and chlorine levels were increased to 1.75 mmol/L and 122 mmol/L, respectively. Leukopenia was still notable on laboratory results (white blood count, 2.65*10^9^/L; neutrophil count, 0.78*10^9^/L). The patient had no neurological deficits and was discharged on July 23. Minocycline (100 mg p.o. twice a day [b.i.d.]) was continued for two weeks after discharge.

According to the severity and rarity of the case, secondary immunodeficiency was suspected. We measured serum immunoglobulin levels and found that all Ig classes were low: IgA, < 0.26 g/L (normal, 0.7–4 g/L); IgG 6.4 g/L (normal, 7–16 g/L); IgM < 0.174 g/L (normal, 0.4–2.3 g/L). Flow cytometry of the patient’s peripheral blood lymphocytes revealed undetectable levels of peripheral B cells (0.0%; reference, 5–18%), CD4-positive T-cell lymphopenia (22.5%; reference, 27–51%), a CD4-positive T-cell count of 754/μL, a CD8-positive T-cell count of 2134/μL, and an inverted CD4-positive/CD8-positive cell ratio (0.4; reference, 0.7–2.8). GS was eventually diagnosed based on thymoma history and the patient's susceptibility to opportunistic infections due to immune deficiency syndrome.

Follow-up evaluation of this GS patient two months after discharge demonstrated good status with normal CSF cell counts and negative findings on NGS of CSF. However, hypogammaglobulinaemia and leukopenia persisted. On August 13, 2021, we conducted a follow-up by telephone; the patient was in good condition except for occasional acute sinusitis and mild cough symptoms, which were relieved by anti-infective therapy every time. She had not acquired any common autoimmune phenomena and had never been treated with immunoglobulin replacement treatment.

## Discussion and conclusions

An estimated 10% of the patients with adult-onset hypogammaglobulinaemia have a thymoma, and among patients with a thymoma, 5% will have hypogammaglobulinaemia [4]. GS is an adult-onset immunodeficiency characterized by thymoma, hypogammaglobulinaemia, low or absent B‑cells, and an inverted CD4+/CD8+ T‑cell ratio [2].

Patients with GS are most commonly between the ages of 40 and 70 years [5]. It has been reported in foreign literature that the number of male and female patients is equal, but there are more female patients in China. In 1999, the World Health Organization (WHO) and the International Federation of Immunology Societies classified GS as an independent cause of primary immunodeficiency. The pathogenesis of immunodeficiency in GS remains unclear and affects both humoral and cellular immunity. There is evidence of a bone marrow defect that produces B cell maturation detention in the pre-B stage, which results in B cell lymphopenia with hypogammaglobulinaemia. GS is frequently associated with haematological disorders. The presence of B and T cell lymphopenia, pre-B cell arrest, pure red cell aplasia, neutropenia, and eosinopenia in many GS cases also suggests that the basic defect may be in the bone marrow. Because of the resultant immunodeficiency, patients with GS may develop severe bacterial, fungal, viral, and other opportunistic infections [6].

UU is found among the normal vaginal flora in a considerable proportion of asymptomatic women; however, adult CNS infection of UU is extremely rare [7]. UU is a member of the Mycoplasmataceae family. The isolation of UU from the cervix alone does not mean pathogenicity. The pathogenic mechanism of UU may be that when the body’s immune response is low or the mucosa is damaged, UU embedded on the cell surface multiplies to compete with the host cell for nutrients, resulting in abnormal chromosomes of the host cell and affecting the synthesis of protein and DNA. Individuals with hypogammaglobulinaemia appear to be more susceptible to the colonization of mucosa with ureaplasmas [8]. We speculate that UU meningitis in this patient is related to mucosal destruction by functional endoscopic sinus surgery. However, we did not culture genitourinary tract secretions, and the source of paranasal sinus UU remained unclear.

Colonization and CNS infection with UU are not rare in newborns or premature infants. However, only two adult cases of UU CNS infection have been reported thus far. One patient developed UU meningitis after a complicated kidney transplant [9], and the other patient who had hypogammaglobulinaemia developed a brain abscess caused by UU after rituximab therapy [7].

Most neurology departments do not have a routine microbiological examination for CNS mycoplasmal infection. NGS is increasingly used for the clinical diagnosis of CNS in China [10]. First-generation sequencing technology, also known as Sanger sequencing, can only sequence a single DNA fragment at a time; it is costly and time-consuming for multiple variants across targeted areas of the genome. PCR can only detect known sequences. In contrast, NGS sequences millions of fragments simultaneously per run; this high-throughput process means that hundreds to thousands of genes can be sequenced at a time. Meanwhile, a higher sequencing depth enables higher sensitivity. NGS is also a hypothesis-free approach that does not require prior knowledge of sequence information. However, the library preparation methods and reagents used in NGS technology may produce low-level but detectable sequencing errors. Many laboratories use PCR and Sanger sequencing to validate the results of NGS [11].

To date, there is no consensus on the optimal treatment of UU meningitis in adults. Because of UU's lack of cell walls and because it is not sensitive to B-lactam antibiotics (such as penicillins and cephalosporins), treatment depends on antibiotics affecting protein or DNA synthesis. Macrolides, tetracyclines and quinolones are empirically used in UU infection of the genitourinary tract. Due to the low CNS penetration, macrolides are not a valid treatment option for UU meningitis. Although levofloxacin can penetrate the blood–brain barrier [12], several studies have reported that UU has high resistance rates to levofloxacin in China [13, 14], and in our case the drug did not significantly relieve the patient’s symptoms. However, we still considered levofloxacin to have some effect on UU, and we continued to administer the medication after UU DNA in the CSF was confirmed by NGS. When minocycline, which is highly concentrated in the CSF [15] and sensitive to UU [16], was added, our patient's neurological conditions were significantly improved.

Whether thymoma resection should be performed for GS patients is still controversial. Although it can relieve the local symptoms caused by thymoma compression and prevent the local infiltration and distant metastasis of thymoma, there are no reported cases of reversal of immunodeficiency after thymoma resection [17]. Our patient underwent thymectomy many years ago, which seemed to confirm that the operation did not improve immune function. Immunoglobulin replacement treatment has been reported to improve infection control. A retrospective study of immunoglobulin therapy for GS reported that 23 of 30 patients had remission of bacterial lung infection [18]. Other scholars believe that intravenous immune globulin (IVIG) can reduce the risk of infection in GS patients by approximately 37.5% [19]. Our patient had never experienced any severe infection requiring hospitalization except meningitis at this time. During follow-up three years after discharge, she was in good condition except for occasional acute sinusitis and mild cough. IVIG is still in the self-pay market without reimbursement. Our patient’s symptoms had improved following the use of anti-infective agents, so she had not considered receiving IVIG to treat or prevent infection.

In conclusion, patients with GS usually have severe or recurrent opportunistic infections. UU can produce CNS infections in immunocompromised hosts, especially when the mucosa is damaged. To our knowledge, this is the first case report in the literature that describes UU meningitis in a patient with GS. With this report, we wish to increase physician awareness that for patients who develop unusual and severe infections, NGS technology has a more rapid and sensitive method of detection Further, levels of T cell subsets, B cells, and immunoglobulin should be measured at an early stage. The diagnosis of GS should be considered in patients presenting with unexplained antibody deficiency and thymoma. Timely aetiological diagnosis and aggressive treatment of infection are necessary for the long-term survival of these patients.

## Data Availability

The datasets used and/or analysed during the current study are available from the corresponding author on reasonable request.

## Abbreviations

UU: *Ureaplasma urealyticum*
CNS: Central nervous system
GS: Good's syndrome
NGS: Next-generation sequencing
CVID: Common variable immunodeficiency disease
CSF: Cerebrospinal fluid
WHO: World Health Organization
DNA: Deoxyribonucleic acid
ENT: Ear, nose, and throat
VAS: Visual analogue scale
CT: Computed tomography
MRI: Magnetic resonance imaging
HIV: Human immunodeficiency virus
ANCA: Anti-neutrophil antibodies
PCR: Polymerase chain reaction
IVIG: Intravenous immune globulin
