# Correction to: Case report of Ureaplasma urealyticum meningitis in a patient with thymoma and hypogammaglobulinaemia

**DOI:** 10.1186/s12879-021-06885-z

**Published:** 2021-11-22

**Authors:** Ting Zhang, Haiyan Li, Shuping Hou, Huanxin Yu, Wei Yue

**Affiliations:** 1 Clinical College of Neurology, Neurosurgery and Neurorehabilitation, Tianjin Medical University, Tianjin, China; 2 Present Address: Department of Neurology, Tianjin Key Laboratory of Cerebrovascular and Neurodegenerative Diseases, Tianjin Huanhu Hospital, Tianjin, China; 3 Department of Otolaryngology, Tianjin Key Laboratory of Cerebrovascular and Neurodegenerative Diseases, Tianjin Huanhu Hospital, Tianjin, China; 4 Department of Dermatology, Tianjin Medical University General Hospital, Tianjin, China

## Correction to: BMC Infect Dis (2021) 21:1142 https://doi.org/10.1186/s12879-021-06831-z

Following the publication of the original article [1], an error was notified in the authors’ list affiliation.

The authors’ list currently read:

Ting Zhang^1^, Haiyan Li^3^, Shuping Hou^4^, Huanxin Yu^3^ and Wei Yue^1,2,*^

The author’s list should read:

Ting Zhang^1,2^, Haiyan Li^3^, Shuping Hou^4^, Huanxin Yu^3^ and Wei Yue^1,2,*^

The original article [1] has been corrected.
